# First-Principle Studies of the Vibrational Properties of Carbonates under Pressure

**DOI:** 10.3390/s21113644

**Published:** 2021-05-24

**Authors:** Yurii N. Zhuravlev, Victor V. Atuchin

**Affiliations:** 1 Institute of Basic Sciences, Kemerovo State University, 650000 Kemerovo, Russia; zhur@kemsu.ru; 2 Research and Development Department, Kemerovo State University, 650000 Kemerovo, Russia; 3 Laboratory of Optical Materials and Structures, Institute of Semiconductor Physics, SB RAS, 630090 Novosibirsk, Russia; 4 Department of Applied Physics, Novosibirsk State University, 630090 Novosibirsk, Russia

**Keywords:** calcite, dolomite, aragonite, pressure, vibrational spectroscopy, hydrostaticity, first-principle calculation

## Abstract

Using the density functional theory with the hybrid functional B3LYP and the basis of localized orbitals of the CRYSTAL17 program code, the dependences of the wavenumbers of normal long-wave *ν* vibrations on the *P*(GPa) pressure *ν*(cm^−1^) = *ν*_0_ + (*dv*/*dP*)·*P* + (*d*^2^*v*/*dP*^2^)·*P* and structural parameters *R*(Å) (*R*: *a*, *b*, *c*, *R*_M-O_, *R*_C-O_): *ν*(cm^−1^) = *ν*_0_ + (*dv*/*dR*) − (*R* − *R*_0_) were calculated. Calculations were made for crystals with the structure of calcite (MgCO_3_, ZnCO_3_, CdCO_3_), dolomite (CaMg(CO_3_)_2_, CdMg(CO_3_)_2_, CaZn(CO_3_)_2_) and aragonite (SrCO_3_, BaCO_3_, PbCO_3_). A comparison with the experimental data showed that the derivatives can be used to determine the *P* pressures, *a*, *b*, *c* lattice constants and the *R*_M-O_ metal-oxygen, and the *R*_C-O_ carbon-oxygen interatomic distances from the known Δ*ν* shifts. It was found that, with the increasing pressure, the lattice constants and distances *R* decrease, and the wavenumbers increase with velocities the more, the higher the *ν*_0_ is. The exceptions were individual low-frequency lattice modes and out-of-plane vibrations of the *v*_2_-type carbonate ion, for which the dependences are either nonlinear or have negative *dv/dP* (positive *dv*/*dR*) derivatives. The reason for this lies in the properties of chemical bonding and the nature of atomic displacements during these vibrations, which cause a decrease in *R*_M-O_ and an increase in *R*_C-O_.

## 1. Introduction

Carbonates are among the most common minerals in the earth crust, and they play a key role in the dynamics of many geological processes [1]. How spectral characteristics of the infrared absorption and Raman scattering depend on pressure is of considerable research interest, since it allows us to study the behavior of carbonates in extreme natural conditions, including the deep mantle of the Earth. Knowing a physical and chemical property of carbonates, as the main carriers of carbon subduction in the Earth interior, is crucial for understanding the deep carbon cycle and related geological processes on the planet [2].

Vibrational modes of carbonates, among other materials with complex ions, are of particular interest since they are a convenient model for studying the “internal” modes of ions, along with the “external” modes that arise due to intragranular movements, complex ion rotations and metal cation vibrations [3]. There is another important reason for studying the vibrational modes of carbonates at a high pressure. It is known that the ruby fluorescence scale is widely recognized as the most reliable and convenient calibration for determining the pressure value in the experiments at high temperatures in a diamond anvil cell [4]. The use of such pressure scale requires a specified laser set up and a spectrometer to actuate and measure the corresponding fluorescence lines. However, this classical optical system is not always available in infrared spectroscopic experiments. It would be useful to develop a secondary pressure reference based on the calibrated shift of the IR-active modes of another convenient crystal phase, for which the internal ${\mathrm{CO}}_{3}^{2-}$ vibration modes of ions in carbonates can be chosen. In particular, the possibility of using carbonate crystals as pressure sensors was exemplified by the selection of calcite and dolomite [5]. The specific features of carbonates vibrational spectra are the segregation of vibration modes into lattice (0–400 cm^−1^) and intramolecular modes of four types: deformations in the carbonate ion plane (*ν*_4_, 650–720 cm^−1^), deformations outside the plane (*ν*_2_, 840–910 cm^−1^), symmetric stretching (*ν*_1_, 1000–1100 cm^−1^) and asymmetric stretching (*ν*_3_, 1350–1600 cm^−1^) [6].

The experimental measurements of the infrared absorption spectra (IRS) of magnesite (MgCO_3_) within the frequency range of 100–1800 cm^−1^, depending on the pressure up to 28.8 GPa, were performed in [7]. The large Gruneisen parameter values for the translational modes were explained there by the fact that the pressure-induced changes in the interatomic distances in the [MgO_6_] octahedra are significantly greater than in the ${\mathrm{CO}}_{3}^{2-}$ ions, which remained practically rigid. In [8], the behavior of the infrared and Raman spectra (RS) of magnesite in the pressure range 0–50 GPa was studied within the density functional theory (DFT), taking into account the third-order perturbation. Further, high pressure Raman spectroscopy up to 27 GPa was used to study a single crystal of magnesite in [9], and it was shown that the wavenumbers of all single-degenerate modes increased almost linearly with the increasing pressure. The first-principle studies of thermodynamic and elastic properties of magnesite under pressure were held in the local approximation (LDA) DFT in [10]. Raman spectroscopy, in combination with X-ray diffraction and DFT calculations, was used in [11] to establish the magnesite phase stability.

The behavior of natural calcite minerals at a high pressure up to 75 GPa was studied using Raman laser spectroscopy and theoretical DFT calculations in [12]. The behavior of otavite (CdCO_3_) at the pressure up to 23 GPa was studied using RS and DFT calculations with the PBE gradient functional in [13]. It was shown that the calcite-type structure remains stable to a pressure of at least 19 GPa. A combined IRS and high-pressure RS study of a natural CaMg_0.98_Fe_0.02_(CO_3_)_2_ dolomite sample was carried out in [14]. Here, the data obtained in the framework of DFT with the LDA and PBE exchange-correlation functionals and the plane wave basis were used to interpret the infrared and Raman spectra measured in the ranges of 500–2000 cm^−1^ and 100–1200 cm^−1^, respectively. In [15], the studies of the properties of a natural dolomite sample without iron admixture were carried out for pressures up to 40 GPa and the far IR band.

The infrared spectrum of cerussite (PbCO_3_) in the aragonite structure for pressures up to 41 GPa was measured in a diamond anvil cell in [16]. Infrared spectroscopy was used to study the vibrational spectrum of viterite (BaCO_3_) at the pressure up to 8 GPa to determine the boundaries of a metastable trigonal phase [17]. It was shown that all the carbonate group vibrational modes up to 7 GPa pressure, with the exception of the out-of-plane ν_2_ deformation vibration, shift towards higher wavenumbers. The phase behavior of strontianite (SrCO_3_) was studied up to 78 GPa experimentally using RS and theoretically using the PBE functional in [18]. In [19], joint studies were carried out using the methods of infrared absorption and Raman scattering on a sample of a synthetic strontianite crystal, providing the basis for a pressure-temperature phase diagram.

Of considerable interest is the comparative analysis of the spectroscopic characteristics of carbonates of various structural types. The aragonite, magnesite and dolomite Raman spectra were studied under pressure up to 25 GPa in [20]. It was shown that the changes in wavenumbers under pressure are greater for lattice modes than those for intramolecular ones, as evidenced by the mode anharmonic parameters. The thermodynamic and thermoelastic properties of magnesite, calcite, aragonite, dolomite and siderite were predicted using IRS and RS [21]. The pressure- and temperature-induced frequency shifts of the Raman vibrational modes of the natural aragonite, calcite, dolomite and magnesite minerals were obtained [22]. Finally, the IRS and RS were measured for aragonite, stronzianite, cerussite and viterite [6], where it is shown that Gruneisen isobaric and isothermal parameters are in the range of 0.9–3.5 for the lattice vibrational modes below 350 cm^−1^, and less than 0.4 for the ${\mathrm{CO}}_{3}^{2-}$ internal modes above 650 cm^−1^.

This work is aimed at the systemic study, by computer modeling methods, of the behavior of the vibrational spectra of simple and double metal carbonates under pressure. Previously, such combined approach was successfully applied for the exploration of structural, electronic and spectroscopic characteristics of crystal materials related to different chemical classes [23,24,25,26,27,28]. The main regularities of changes in the parameters of the vibrational modes, the quantitative dependences of the wavenumbers on pressure and structural parameters of carbonates, as well as the possibility of determining the structural characteristics from the known wavenumber shifts, will be established. Such dependences are valuable in studying the characteristics of carbonate crystals by noncontact nondestructive optical methods [29,30,31].

## 2. Calculation Method

The study of metal carbonates spectroscopic parameters as a function of pressure is based on the first principles of the Hartree-Fock theory (HF) and the density functional theory that are well combined in the CRYSTAL17 program code [32,33]. The calculations used the B3LYP hybrid functional, which includes the 20% HF exchange with the BECKE exchange functional [34] and the LYP correlation functional [35]. The basic functions were chosen as a linear combination of localized Gaussian-type atomic orbitals. Full-electron basis sets for carbon, oxygen, magnesium and calcium atoms from [36] were used. The POB-DZVP basis was used for zinc atoms [37]. For strontium and barium, the pseudopotential basis sets from [38] were used, and for lead—those from [39]. The inverse space is discretized using a Monkhorst-Pack [40] grid with 216 independent ***k***-points in the irreducible part of the Brillouin zone for trigonal crystals and 64 points—for orthorhombic ones. The accuracy of self-negotiation procedure was above 10^−9^ au. (1 au. = 27.21 eV). The wavenumbers of harmonic vibrations of the lattice atoms were calculated using the FREQCALC procedure [41,42]. The harmonic frequencies of phonons at Γ point (***k*** = 0, the center of the Brillouin zone) were obtained from the diagonalization of the mass-weighted matrix of the second energy derivatives, with respect to atomic displacements [43].

To describe the wavenumbers dependence on the *P* (GPa) pressure, the Gruneisen mode parameter is used [7]: $\gamma_{i}=\left(B_{0}/\nu_{i}\right)\left(\partial \nu_{i}/\partial P\right)$, where *ν_i_* is the wavenumber of the *i*-vibrational mode (cm^−1^), *V* is the unit cell volume (Å^3^), *B*_0_ is the isothermal volume compression modulus (GPa) determined from the Birch-Murnaghan constitutive equation [44]: $P\left(V\right)=\frac{3B_{0}}{2}\left(x^{-7}-x^{-5}\right)\left(1+\frac{3}{4}\left(B_{1}-4\right)\left(x^{-2}-1\right)\right),\;x={\left(V/V_{0}\right)}^{1/3}$,$\;B_{1}={\left(\partial B/\partial P\right)}_{T}$, the first derivative of the pressure modulus being *x* = 1. The wavenumber pressure derivatives were calculated numerically from the quadratic interpolation $\nu \left(P\right)=\nu_{0}\left(P=0\right)+(d\nu /dP)\cdot P+(d^{2}\nu /dP^{2})\cdot P^{2}$. The accuracy of this procedure is controlled by the correlation coefficient $K=\sqrt{\overset{N}{\underset{i=1}{\sum }}{\left(y_{i}^{fit}-\bar{y^{fit}}\right)}^{2}/\overset{N}{\underset{i=1}{\sum }}{\left(y_{i}^{data}-\bar{y^{data}}\right)}^{2}}$, where the average value of $\bar{y}=\frac{1}{N}\overset{N}{\underset{i=1}{\sum }}y_{i}$. The temperature in these calculations was not taken into account and, by default everywhere, it is equal to absolute zero.

## 3. Crystal Structure and Pressure

To determine the parameters of carbonate crystal structure, a complete optimization for lattice constants and atomic positions coordinates was performed. The starting values used are the data known from the literature for magnesite [9], smithsonite (ZnCO_3_) [45], otavite [46], dolomite [47], minocordite (CaZn(CO_3_)_2_) [48], CdMg(CO_3_)_2_ [49], strontianite, cerussite and viterite [50]. The obtained lattice constants and interatomic distances (Table 1) are in a good agreement with the experimental values. Deviations do not exceed 3% for natural minerals and 1% for synthetic crystals [51].

To study the effect of the *P* pressure, hydrostatic compression was set in the range of 0–10 GPa, and then the resulting structure was optimized while maintaining the *V* cell volume. The obtained dependences *V*(*P*) were used to determine the *V*_0_, *B*_0_, *B*_1_ state equation parameters, the *a*(*P*), *b*(*P*), *c*(*P*) lattice constant dependences and the interatomic distances: *R*_M-O_(*P*)—for metal-oxygen, and *R*_C-O_(*P*)—for carbon-oxygen. The linear compression modules $B_{x}=-x\cdot \partial P/\partial x\;\left(x:a,\;b,\;c,\;R_{M-O},\;R_{C-O}\right)$ were determined from the obtained dependences. The derivative was calculated by linear interpolation. The interatomic distances and lattice constants can then be obtained by the formula $x\left(P\right)=x_{0}\left(1-P/B_{x}\right)$. The parameters of state equation and linear compressibility modules obtained this way are shown in Table 2.

**Figure 1:**
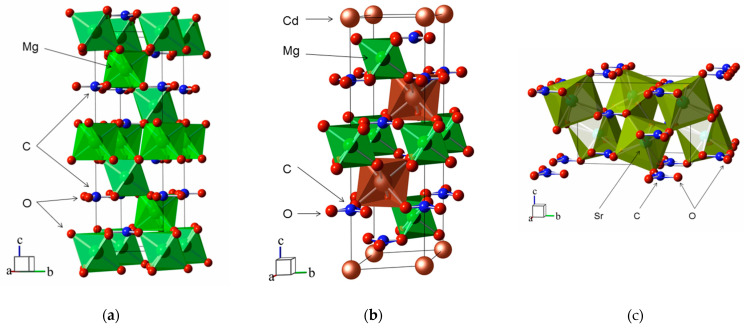
Crystal structures of (**a**) MgCO_3_ (calcite), (**b**) CdMg(CO_3_)_2_ (dolomite) and (**c**) SrCO_3_ (aragonite). The unit cells are outlined. Lone atoms, except for those in the unit cells, are omitted for clarity.

The typical crystal structures of carbonates from the calcite, dolomite and aragonite families are shown in Figure 1. Carbonates of the calcite family MCO_3_ (M: Mg, Zn, Cd) belong to the $R\bar{3}c$ space group and contain two formula units (*Z* = 2) in a unit cell, which volume increases with the atomic radius growth of the *R*_M_ cation [52] as *V*_0_(Å^3^) = 9.919 + 52.21·*R*_M_. As the pressure increases, the *a*, *c* lattice constants and interatomic distances decrease linearly. The *B_a_*, *B_c_* moduli in MgCO_3_ that characterize this decrease for the same pressure range are consistent with the experimental data [53]. In rhombohedral lattices, the M^2+^ cations and ${\mathrm{CO}}_{3}^{2-}$ anions are arranged in layers perpendicular to the *c* axis, and, so, the compressibility along the *a* axis is almost three times less than that along the *c* axis. The atoms in the ${\mathrm{CO}}_{3}^{2-}$ groups are bound together by strong internal C-O bonds and weaker M-O bonds in octahedra [MO_6_], and, so, the former are practically incompressible and *R*_M-O_ decrease quite rapidly with the increasing pressure.

Double carbonates with a dolomite structure belong to the $R\bar{3}$ (*Z* = 2) space group. This structure consists of alternating layers of [CaO_6_] and [MgO_6_] octahedra arranged sequentially along the *c* axis and separated by the ${\mathrm{CO}}_{3}^{2-}$ plane groups in the parallel *ab* plane. There is also a significant anisotropy of compressibility along the axes *a* and *c*: *B_a_*/*B_c_* = 3.1 (3.0 in [47]). The unit cell volume in double carbonates grows with an increase in the arithmetic mean cation radius; however, as in calcites, there are no ordinary change patterns in the volume and linear compressibility moduli.

SrCO_3_, PbCO_3_ and BaCO_3_ carbonates have an aragonite-type structure and belong to the space group *Pmcn* (Z = 4). The orthorhombic crystal structure consists of coplanar ${\mathrm{CO}}_{3}^{2-}$ triangles parallel to the *ab* plane and the [MO_9_] (M^2+^: Sr, Pb, Ba) polyhedra located along the *c* axis. The cation layers in an approximately hexagonal tightly packed structure alternate with anion layers. There is also a significant compressibility anisotropy: it is minimal along the *a* axis and maximal along the *c* axis. In BaCO_3_, the *b* constant dependence has a nonlinear character and is described by the quadratic function $b\left(Å\right)=8.933-0.042\cdot P+0.004\cdot P^{2}$ with the correlation coefficient of 0.998. For the average distances <M-O> and <C-O>, the linear dependence is preserved and the rate of their decrease with the increasing pressure is −0.013 Å/GPa and −0.001 Å/GPa (−0.013 and −0.003 Å/GPa as in [54]), respectively.

## 4. Vibrational Spectra under Pressure

The decrease in the interatomic distances under hydrostatic pressure is accompanied by an increase in the wavenumbers of vibrational modes. Therefore, the energy of zero vibrations $E_{0}=\overset{N}{\underset{i=1}{\sum }}h\nu_{i}/2$ grows with the increasing pressure and this dependence can be described by $E_{0}\left(\mathrm{kJ}/\mathrm{mol}\right)=E_{0}+(dE_{0}/dP)\cdot P+(d^{2}E_{0}/dP^{2})\cdot P^{2}$, which coefficients, in terms of a *Z* formula unit, are given in Table 3. There is a linear correlation between the zero vibrations energy and the *M* cation atomic mass [55] (the average for cations in double carbonates): *E*_0_ (kJ/mol) = 50.797 − 0.041·*M*.

The M-O and C-O interatomic distances change differently with the increasing pressure, and, so, the wavenumbers of the ${\mathrm{CO}}_{3}^{2-}$ intramolecular vibrations can be expected to vary slightly, compared to the wavenumbers of lattice vibrations. Lattice vibrations are divided into translational (T) vibrations involving cations and anions and rotational (L) vibrations for anions. For the center of Brillouin zone (Γ), the crystal vibrational modes can be classified by irreducible representations of the point symmetry group. For crystals with a calcite structure, the total vibrational representation is decomposed into irreducible ones: Γ_tot_ = *A*_1g_(RS) + 3*A*_1u_ + 3*A*_2g_ + 3*A*_2u_(IRS) + 4*E*_g_(RS) + 6*E*_u_(IRS), where IRS means modes active in the infrared spectrum, and RS stands for modes active in the Raman spectrum. For the dolomite structure, the expansion of the vibrational representation has the form: Γ_tot_ = 4*A_g_*(RS) + 6*A_u_*(IRS) + 4*E_g_*(RS) + 6*E_u_*(IRS); respectively, for aragonite: Γ_tot_ = 9*A_g_* + 6*B*_1g_(RS) + 9*B*_2g_(RS) + 6*B*_3g_(RS) + 6*A*_u_ + 9*B*_1u_(IRS) + 6*B*_2u_(IRS) + 9*B*_3u_(IRS). The numbers before the irreducible representations indicate the number of vibrational modes of this type.

In calcites, the *A*_2u_ (*A*_u_ in dolomite) symmetry modes with the polarization along the *c* axis must be more pressure-dependent than the *E*_u_ symmetry modes with the polarization perpendicular to this axis. In aragonites, the *B*_2u_ vibrational modes have the polarization ***E***||***a***, *B*_3u_-***E***||***b***, *B*_1u_-***E***||***c***. Of particular interest are the vibrational modes, which wavenumbers do not increase with the increasing pressure, but rather decrease. They have a negative pressure derivative and a negative Gruneisen parameter. According to the literature, these include the ν_2_-type mode of the *A*_u_ [14] and *A_g_* [22] symmetries of dolomite, the *B*_1u_, *B*_3u_, *A*_g_ and *B*_2g_ modes of aragonite [56], viterite [17], cerussite [16] and strontianite [19]. The low-wavenumber lattice vibrations in dolomite also have negative mode parameters [8].

**Figure 2:**
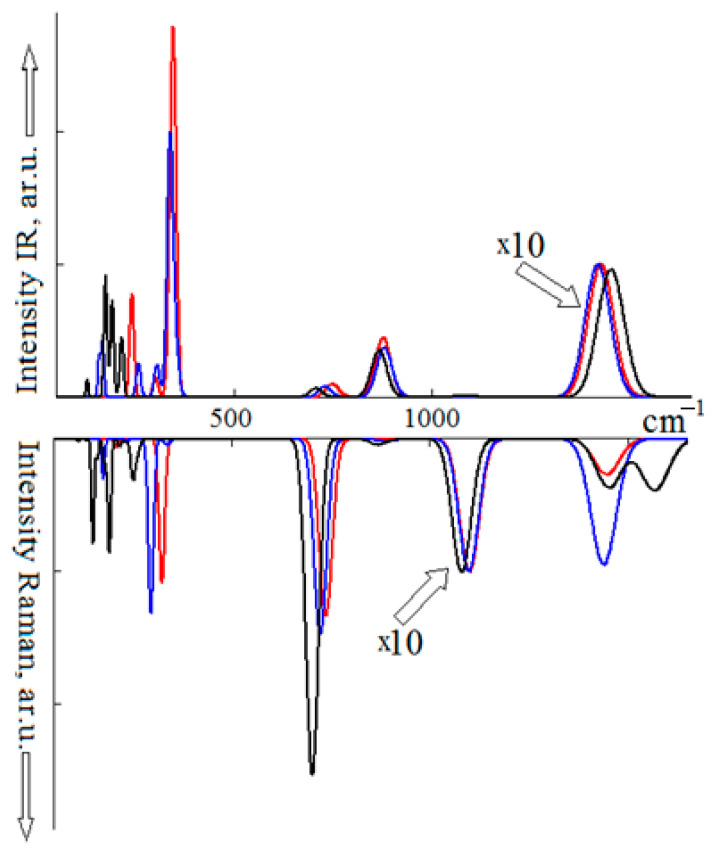
Infrared (**top**) and Raman (**bottom**) spectra of MgCO_3_ (red), CaMg(CO_3_)_2_ (blue) and SrCO_3_ (black). ×10 means that the intensity of the corresponding line should be increased 10 times.

The infrared and Raman spectra obtained by the Gaussian broadening of the normal MgCO_3_, CaMg(CO_3_)_2_ and SrCO_3_ long-wave vibrational modes are shown in Figure 2. For other carbonates, the spectra have a qualitatively similar form [51]. The MgCO_3_ IRS is dominated by an intense (assumed to be 100%) band formed by the *v*_3_ *E*_u_-symmetry mode with the wavenumber of 1423 cm^−1^. The other two intramolecular modes, *v*_2_ and *v*_4_, have significantly lower intensities of 4 and 1%, and wavenumbers of 874 and 745 cm^−1^, respectively. In CaMg(CO_3_)_2_, the positions of these bands maxima do not change significantly: 1415 (*E*_u_), 877 (*A*_u_) and 726 cm^−1^ (*E*_u_). Nor does their intensity change. The allowed *v*_1_ type vibration has a near-zero intensity. In SrCO_3_, due to a decrease in the anion symmetry, the number of vibrational modes increases, and it does not affect the *v*_3_ maxima position: 1455 (*B*_3u_) cm^−1^, 1439 (*B*_2u_) cm^−1^; *ν*_2_: 864 (*B*_1u_) cm^−1^, *v*_4_: 705 (*B*_3u_) cm^−1^.

In the Raman spectra of MgCO_3_, CaMg(CO_3_)_2_ and SrCO_3_, the *v*_1_ vibration intensity (taken as 100%) dominates, having the wavenumbers of 1099 (*A*_1g_), 1097 (*A*_g_), 1079 (*A*_g_) cm^−1^, respectively. Against this background, other fluctuations are less noticeable. So, for *v*_4_, the intensity in MgCO_3_ equals 13%, in CaMg(CO_3_)_2_—15% and in SrCO_3_—11%. The *v*_3_ band in strontianite has two maxima at 1450 (*B*_3g_) and 1565 cm^−1^ (*B*_2g_) with intensities of 4%; one maximum at 1437 cm^−1^ in dolomite and 1444 cm^−1^ in magnesite. As for the *v*_2_ band, its intensity in all carbonates does not exceed 1%, and the maximum is in the region of ~870 cm^−1^.

The values of the *ν*_0_ wavenumbers and their pressure derivatives *dν*/*dP*, *d*^2^*ν*/*dP*^2^ are summarized in Table 4, Table 5 and Table 6. The Gruneisen mode parameter γ_i_ can easily be obtained using the data from these tables and the *B*_0_ values from Table 2. Further, in Table 4, Table 5 and Table 6, the *d*^2^*P*/*dν*^2^ derivatives are given, with the help of which, along with the (*dν*/*dP*)^−1^ value, it is possible to calculate the pressure from a known wavenumber: $P\left(v\right)=\left(dP/dv\right)\cdot \left(v-v_{0}\right)+\left(d^{2}P/dv^{2}\right)\cdot {\left(v-v_{0}\right)}^{2}$. The *dv*/*da* derivatives are also given for calculating the *a* lattice constant: $a\left(v\right)=a_{0}+\left(da/dv\right)\cdot \left(v-v_{0}\right)$ or the wavenumber: $v\left(a\right)=v_{0}+\left(dv/da\right)\cdot \left(a\;a_{0}\right)$. To calculate other (*b*, *c*, *R*_M-O_, *R*_C-O_) geometric parameters of the lattice, the fact that there are linear dependences between them under pressure will be used. For example, for aragonite [56] in the 0–15 GPa range, the following relations are fulfilled: *b*(Å) = *b*_0_ + 1.325·(*a* − *a*_0_) (0.997), *c*(Å) = *c*_0_ + 1.824·(*a* − *a*_0_) (0.995). Therefore, the *db*/*da*, *dc*/*da*, *dR*_M-O_/*da*, *dR*_M-O_/*da* derivatives can be calculated and, consequently, the necessary *dν*/*dR* = (*dν*/*da*)/(*dR*/*da*) can be determined. For example, to calculate the derivative of *dc*/*da*, the data in Table 1 and Table 2: *dc*/*da* = (*c*_0_/*a*_0_)·(*B_a_*/*B_c_*) are needed. For MgCO_3_, this method gives the value of 7.378, and in the direct calculation—that of 7.376.

**Figure 3:**
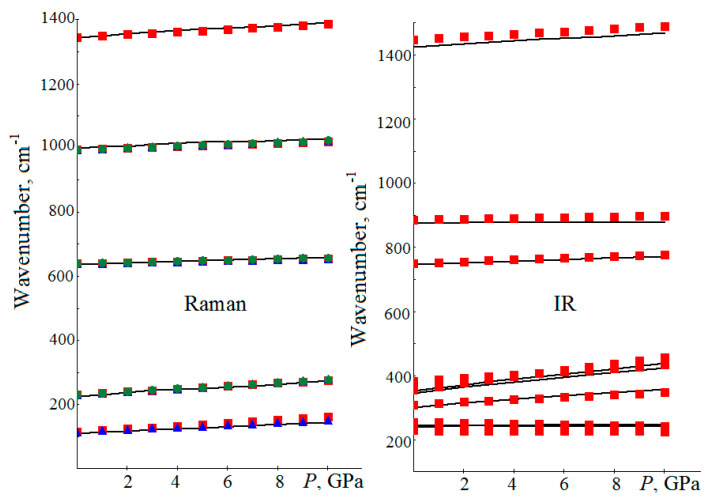
Calculated (the calculation is solid lines, squares, circles and triangles—the experiment) dependences of MgCO_3_ vibrational modes wavenumbers on the *P* pressure, active in the Raman spectrum (on the left, squares—the data from [20], triangles from [22], circles from [9]) and the infrared spectrum (on the right, squares—the data from [7]).

The pressure dependences of magnesite wavenumbers for five vibrational modes active in the Raman spectrum and eight modes active in the infrared spectrum are shown in Figure 3. The wavenumbers of all modes observed in RS continuously and almost linearly increase with the increasing pressure. The values of the *dν_i_*/*dP* derivatives obtained in this research (Table 4) are in a satisfactory agreement with the available experimental data [9,22,53]. The discrepancies do not concern the curve shapes, they concern only their displacements relative to the calculated and measured *ν*_0_ values. Thus, using the described calculation algorithm and the *ν*(*P*) numerical dependences from [9], for the 0–12 GPa pressure range, *v*_T_(cm^−1^) = 212 + 4.213·*P* − 0.076·*P*^2^ (0.999) is obtained. There is also a satisfactory agreement for the Gruneisen mode parameters obtained earlier experimentally in [20] and theoretically in [10].

Here is an example of calculating an unknown pressure from a known *v* wavenumber. To do this, the *E_g_*(T) MgCO_3_ lattice vibration is chosen. Using the derivatives of Table 4, the following is obtained: *P*(GPa) = (5.541)^−1^·(*ν*_T_ − *ν*_T,0_) + 0.606·10^−3^·(*ν*_T_ − *ν*_T,0_)^2^ (0.994), and for the experimental data from [9]: *P*(GPa) = 0.176·(*ν*_T_ − *ν*_T,0_) + 0.698·10^−3^·(*ν_T_* − *ν*_T,0_)^2^ (0.999). In [9], the Δ*ν*_T_ shift is 28 cm^−1^ at 5.58 (±0.06) GPa and the first theoretical formula yields *P* = 5.53 GPa, the second experimental formula—5.48 GPa. For the *ν*_1_ intramolecular vibration, according to [9], at the specified pressure for the Δ*ν*_1_ = 18 cm^−1^ shift, we obtain it using the theoretical formula 5.54 GPa, while the experimental one yields 5.60 GPa. Thus, the derivative values obtained in Table 4 allow us to calculate the external pressure on the sample according to the known displacement of the wavenumber fairly accurately.

The IRS-active modes can also be used to determine pressures. For this purpose, the data from [7] for the *ν*_3_ intramolecular vibration is used, where for the pressure of 6.2 ± 0.3 GPa, the shift Δν_3_ = 20 cm^−1^ is determined. Using only the first derivatives, the pressure of 4.73 GPa for *dP*/*dν* from [7] can be calculated, the same as 4.23 GPa from [8]. In this research, for the derivative 4.347 cm^−1^/GPa, 4.60 GPa is obtained, and it is less than the experimental data. The quadratic interpolation of experimental values according to the formula *P*(GPa) = 0.325·(Δ*ν*_3_) − 1.489·10^−3^·(Δ*ν*_3_)^2^ yields the value of 6.5 GPa, which is in a satisfactory agreement with the experiment. The exact pressure value can only be obtained for the cubic dependence, which coefficients can be found in [7].

There is a linear relationship between the wavenumbers and pressure, as well as between the lattice geometric parameters and pressure. This means that, between *ν_i_* and the lattice parameters (*a*, *c*, *R*_M-O_, *R*_-C-O_), there is also a linear relationship. For example, for the *ν*_T_ RS mode in MgCO_3_, the relation between its wavenumber and the *a* lattice constant is satisfied: *ν*_T_(cm^−1^) = *ν*_T,0_ − 600·(*a* − *a*_0_). For the experimental values of *a* = 4.595 Å lattice constant [53] at the pressure of 4.20 GPa and *a*_0_ = 4.628 Å in the absence of pressure, the formula yields Δ*ν*_T_ = 19.8 cm^−1^ (18.1 cm^−1^ in [9]). At the pressure of 6.1 GPa in [44] Δ*a* = −0.042 Å, and, so, for the *ν*_3_ IRS active mode, the result is Δ*ν*_3_ = 21.1 cm^−1^. In the [7] experiment at 6.2 GPa, this shift is 20 cm^−1^. There is also an inverse relationship: *a*(Å) = *a*_0_ − 600^−1^·(*ν*_T_ − *ν*_T,0_). For the above experimental data for the *ν*_T_ mode, this formula gives a lattice constant value of 4.593 Å. For the Δ*ν*_1_ shift at the pressure of 4.08 GPa [9], according to *a*(Å) = *a_0_* − 360^−1^·(Δ*ν*_1_) formula, the lattice constant will be equal to 4.606 Å. According to [53], this corresponds to the pressure of 4.2 GPa. Thus, the study of how the Raman scattering and infrared absorption spectra of calcites depend on pressure makes it possible to determine the structural parameters from the known wavenumbers and vice versa. At the same time, to improve the prognosis accuracy, instead of the (*P* = 0) equilibrium theoretical values of the *ν*_0_ wavenumbers and the *a*_0_ lattice constants, it is necessary to use experimental data.

The above-mentioned linear dependences do not work for all vibrations. For the lattice symmetry modes *E_u_*(L) and *A*_2*u*_(L) in MgCO_3_, the *ν*(*P*), *ν*(*R*) dependences have a pronounced nonlinear character. In CdCO_3_, the *dν*/*dP* derivative is negative, while the *dν*/*da* is positive, and it means that the wavenumber *v* decreases with the increasing *P* pressure and the geometric parameters of the *R* lattice decrease. There are no such anomalies in ZnCO_3_. The nonlinear dependences are also observed for the intramolecular out-of-plane *ν*_2_ vibrations in MgCO_3_ and ZnCO_3_. In CdCO_3_, the linear relationship with the correlation coefficient of 0.99 is retained, but the derivatives *dν*/*dP* and *dν*/*da* are equal to −0.25 cm^−1^/GPa, and +40 cm^−1^/Å, respectively.

In the lattice vibrations of the *E*_u_(L) symmetry in MgCO_3_, the metal and carbon atoms move in opposite directions in the *ab* plane, and the oxygen atoms rotate in the *c*-axis direction. For the *A*_2u_(L) symmetry, the metal and carbon atoms move in the opposite directions along the *c* axis, and the oxygen atoms move along the *c* axis synchronously with the carbon atom and rotate in the *ab* plane. For the *ν*_2_ type vibrations, the metal and carbon atoms are displaced along the *c* axis, opposite to the oxygen atoms. Thus, with these fluctuations, there is a decrease in the *R*_M-O_ distances and an increase in the *R*_C-O_ ones.

To explain the anomalous behavior with the appearance of increasing pressure of *E_u_*(L), *A*_2*u*_(L) lattice modes and the *ν*_2_ intramolecular mode, the results of chemical bond parameter calculations are used. With the increasing pressure and decreasing interatomic distances, the chemical binding strength of the atoms in the ${\mathrm{CO}}_{3}^{2-}$ group and the cationic polyhedra changes. The chemical bond can be quantitatively characterized by the amount of *P*_A-B_ atom electron cloud overlap population. The overlap population on the Mg-O line equals 0.034 *e* (*e* is the electron charge), Cd-O is 0.05 *e*, and it does not depend on the metal-oxygen distance. Only in ZnCO_3_, as the distance decreases, the population increases according to *P*_Zn-O_(*e*) = 0.077 − 0.065·(*R*_Zn-O_ −*R*_Zn-O,0_). As the *R*_M-O_ distance decreases, the effective charge of the Mg^2+^, Cd^2+^ and Zn^2+^ cation decreases at the rates of −0.43, −0.43 and −0.88 |*e*|/Å, respectively. Thus, the Coulomb energy of its interaction with oxygen increases in the absolute value in MgCO_3_, and decreases faster in CdCO_3_ than in ZnCO_3_.

There are linear relations between the changes in the distances Δ*R*_C-O_ = *R*_C-O_ − *R*_C-O,0_ and the C-O link population overlap: *P*_C-O_(*e*) = 0.3 + 3.367·Δ*R*_C-O_ in MgCO_3_, *P*_C-O_(*e*) = 0.37 + 2.573·Δ*R*_C-O_ in ZnCO_3_ and *P*_C-O_(*e*) = 0.469 + 1.325·Δ*R*_C-O_ in CdCO_3_. This means that, as the *R*_C-O_ distance increases, the *P*_C-O_ population grows and the chemical binding strength increases. Thus, the negative shifts of *E_u_*(L), *A*_2*u*_(L) and the *ν*_2_ wavenumbers under pressure are explained by the changes in the M-O and C-O chemical bonds.

**Figure 4:**
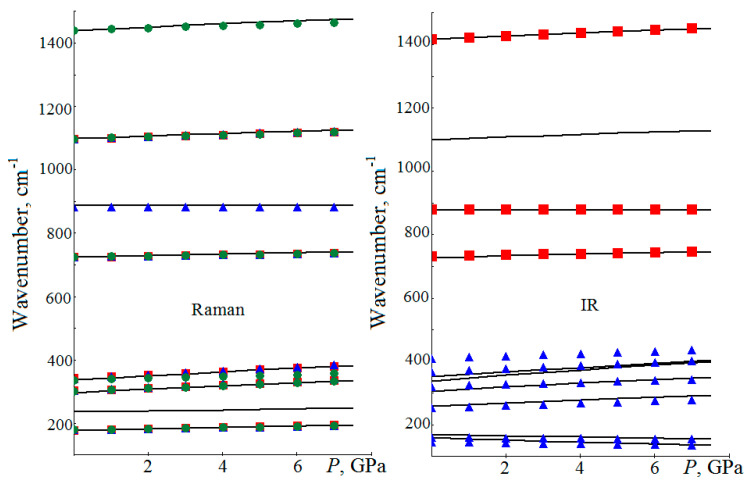
Calculated dependence of lattice and intramolecular vibrational mode wavenumbers (solid lines) of CaMg(CO_3_)_2_ on the *P* pressure, active in the Raman spectrum (left: squares illustrate experimental data from [14], triangles—from [22], circles—from [20]) and the infrared spectrum (right: squares from [14], triangles from [8]).

In the dolomite IRS-absorption (Figure 4), as the pressure increases, the modes above 200 cm^−1^ shift towards larger wavenumbers, and the modes below 200 cm^−1^ shift towards smaller values [8]. In [14], it is shown that for the natural dolomite in the wavenumber range of 500–1800 cm^−1^, the *v*_2_ mode decreases with the increasing pressure at a rate of −0.13 cm^−1^/GPa, while for the *v*_4_ and *v*_3_ modes, the Gruneisen parameter is positive. In RS, with the increasing pressure, the lattice vibrations of *E_g_* and *A_g_* symmetry with wavenumbers 178, 301, 340 cm^−1^ increase with the velocities of 2.83. 5.53. 6.11 cm^−1^/GPa (2.72, 5.40, 6.79 cm^−1^/GPa in this contribution), respectively. For intramolecular vibrations [22], with the increasing pressure, only *v*_2_ decreases and its Gruneisen parameter is 0.01.

In [5], it is proposed to use dolomite as a pressure sensor and, in determining pressure, the empirical formula *P*(GPa) = 0.371·[(*ν*_L_ − *ν*_T_) − (*ν*_L0_ − *ν*_T0_)] is obtained. In our calculations for the 0–8 GPa interval, the coefficient in this formula is 0.392. Here is an example of using this formula. In [22], for lattice vibrations, derivatives *dν*_T_/*dP*, *dν*_L_/*dP* are 2.83 and 5.53 cm^−1^/GPa, according to which, at the pressure of 5 GPa, Δ*ν*_T_ = 14.15 cm^−1^ and Δ*ν*_L_ = 27.65 cm^−1^. Then, according to the given empirical formula, with these shifts, we get the same 5.0 GPa, and 5.3 GPa with the coefficient adopted in this paper. The use of the derivatives from Table 5 for the *ν*_T_, *ν*_L_ experimental values allows us to obtain 5.0 and 4.9 GPa, respectively, and they coincide with the values measured in [22].

For the lattice and intramolecular vibrational modes active in the Raman spectra, except for *v*_2_, the wavenumbers have a linear dependence on the structural parameters. For example, in dolomite, for the *v*_1_ mode, the dependence on the *a* lattice constant has the form of *ν*_1_(cm^−1^) = *v*_10_ − 260·(*a* − *a*_0_) with the correlation coefficient of 0.999. According to [38], for the pressure of 5.59 GPa, the Δ*a* difference is 0.053 Å, which yields a shift of Δ*ν*_1_ = 14 cm^−1^ according to this formula. This value differs by 2 cm^−1^ from the experimental one [22].

In CaMg(CO_3_)_2_, for the IRS-active low-frequency lattice modes of *A_u_*, *E_u_* symmetry, the wavenumbers decrease with the increasing pressure and with the decreasing structural parameters: *ν_A_*_2u_(cm^−1^) = 159 + 342·(*a* − *a*_0_), *ν_Eu_*(cm^−1^) = 168 + 186·(*a* − *a*_0_). The Gruneisen mode parameter for the out-of-plane *v*_2_ vibration in double carbonates is negative. In CaMg(CO_3_), it is equal to 0.07, in CdMg(CO_3_)_2_—0.03 and in CaZn(CO_3_)_2_—0.001. The nonlinear dependences of *ν*_2_(*P*) and *ν*_2_(*R*) are observed for them, when the wavenumber practically does not change with the increasing pressure and decreasing lattice parameters. Due to the special behavior of *A_u_*, *E_u_* lattice symmetry modes and the *v*_2_ mode, they are excluded from Table 5.

According to the data of experimental studies of active vibrations pressure dependence in the Raman spectra [6,18,57,58] the rate of increase in the *v*_4_ wavenumbers grows with the increasing pressure in strontianite, viterite and cerussite as 1.67, 1.7 and 0.72 cm^−1^/GPa. The linear coefficients 1.62, 2.1 and 0.72 cm^−1^/GPa obtained in this research are consistent with these data. The experiments in viterite [6] show a significantly higher rate for the *ν*_1_ mode—3.2 cm^−1^/GPa and for the *ν*_3_ mode—9.05 cm^−1^/GPa. In cerussite, the positions of these bands maxima, practically, do not change, while the velocities become much smaller: 2.4 and 4.4 cm^−1^/GPa. For the *v*_2_ PbCO_3_ mode, the maxima are set at 839, 838 cm^−1^, which, with increasing pressure, shift to lower wavenumber values with velocities of 1.38, 1.61 cm^−1^/GPa. The negative values of the *A_g_* symmetry derivative *v*_2_ mode are also obtained in this research: −2.46 cm^−1^/GPa b SrCO_3_, −1.48 cm^−1^/GPa b BaCO_3_ and −1.37 cm^−1^/GPa in PbCO_3_. The low intensity modes are not given in Table 6.

It follows from Table 6 that the *v*_4_, *v*_1_ and *v*_3_ wavenumbers grow with the increasing pressure at the greater speed, the greater their *v*_0_ values are. There is a linear dependence of wavenumbers on the structural parameters. For example, in BaCO_3_ *Δν*_1*Ag*_(cm^−1^) = −300·*(a* − *a*_0_). According to [45], in viterite at the pressure of 3.94 GPa, the *a* lattice parameter decreases by −0.042 Å, which allows to predict an increase in Δ*ν*_1_ by 12.6 cm^−1^ using this formula. It agrees well with the value of 12.61 cm^−1^, which is obtained in case the experimental derivative *dν*/*dP* from [6] is used. The decrease in the average *R*_Ba-O_ distance at the same pressure is −0.065 Å. Since *dR*_Ba-O_/*da* = 1.189, the *dν*/*dR*_Ba-O_ coefficient will be equal to 252 cm^−1^/Å, which yields the increment *Δν*_1_ = 16.4 cm^−1^: i.e., to calculate the frequency shifts, one can use not only the lattice constants, but also the known interatomic distances.

To verify the values of the derivatives obtained in this research (Table 6), the study [50] is appropriate. In this contribution, for PbCO_3_, the unit cell parameters and wavenumbers under pressure were measured simultaneously by X-ray diffraction and Raman spectroscopy, respectively. Thus, at 6.6 GPa, the *a* lattice constant decreased by −0.0908 Å, the *c* constant—by −0.311 Å, while the value of the *v*_1_ wavenumber increased by 13.4 cm^−1^. For the distance formula at the value *dν*/*da* = 200 cm^−1^/Å, the shift is 18.2 cm^−1^ and 12.9 cm^−1^ for *dv*/*dc* = 42 cm^−1^/Å, i.e., the difference for the *c* constant is less significant. Now let us illustrate the reverse calculation, when the pressure and distances can be determined from the known experimental shift *Δν*_1_. For the quadratic dependence of Table 6, the result of 5.1 GPa is obtained. This is less than the results obtained when the inverse values of the *dv/dP* derivative from [58]—5.82 GPa, from [6]—5.58 GPa, from [57]—5.3 GPa are used. Taking into account the inverse values of the derivatives, the distances will be as follows: *Δa* = 0.067 Å, *Δc* = 0.319 Å. The experimental values of the wavenumber derivative, with respect to the *dν*/*da* lattice constant, can be obtained from the known *a(P)* and *v(P)* dependences by calculating linear coefficients (*dν*/*dP*)·(*dP*/*da*). Thus, according to [58], the first derivative in the product for *v*_1_ is 2.232 cm^−1^/GPa, the second is 75.669 GPa/Å, which gives the target value of 168.8 cm^−1^/Å. For the *c*-axis, this will be 48.2 cm^−1^/Å.

The experimental studies of the pressure dependence of the vibrations active in the IR spectra were performed for SrCO_3_ in [19], BaCO_3_ in [17] and PbCO_3_ in [16]. The maxima of the *v*_1_ band in these carbonates are 1071, 1059, 1051 cm^−1^, respectively, and the *dν/dP* velocities are 2.81, 3.8, 1.8 cm^−1^/GPa. Here are the results of these calculations: 3.08, 3.22, 2.5 cm^−1^/GPa consistent with these data. In the theoretical considerations, there is a good linear relation between the wavenumbers *v*_4_, *v*_1_ and *v*_3_, pressure and structural parameters. For example, for the *B*_2u_ and *B*_3u_ symmetry modes of the *v*_3_ band, in SrCO_3_ Δ*ν*_3*B*2u_(cm^−1^) = −372·*(a − a*_0_), Δ*ν*_3*B*3u_(cm^−1^) = −358·*(a* − *a*_0_). The situation is different with the *v*_2_ type modes. For both *B*_1u_ and *B*_3u_ symmetry vibrations, the wavenumbers dependence on the pressure, as well as on the R distances, is nonlinear. The exception is BaCO_3_, where *ν*_2*B*1u_(cm^−1^) = 869 + 137·*(a* − *a*_0_). For this vibration, carbon atoms in $CO_{3}^{2-}$ move synchronously for all the molecules in the cell in the positive direction of the *c* axis, opposite to the oxygen atoms. In the *B*_3u_ symmetry vibration, the carbon and oxygen atoms shift asynchronously so that the *R*_C-O_ distances decrease for adjacent layers and increase in one layer. Because of this, the intensities of these modes differ by almost two hundredfold.

## 5. Conclusions

The dependence of normal long-wave *v* vibration wavenumbers on the *P* pressure: *ν*(cm^−1^) = *ν*_0_ + (*dν*/*dP*)·*P* + (*d*^2^*ν*/*dP*^2^)·*P*^2^ and *R* (*a*, *b*, *c* lattice constants, *R*_M-O_, *R*_C-O_ interatomic distances) lattice structure parameters was studied using the density functional theory with the hybrid B3LYP function and the basis of localized atomic orbitals of the CRYSTAL17 package for carbonates with the structure of calcite, dolomite and aragonite: *v*(cm^−1^) = *ν*_0_ + (*dν*/*dR*)·(*R* − *R_0_*). The inverse problem can be solved with the help of the obtained values of derivatives using the known frequency shifts Δν = *v* − *ν*_0_ and determining the pressure *P*(GPa) = (*dν*/*dP*)^−1^·Δ*ν* + (*d*^2^*P*/*dv*^2^)·Δ*ν*^2^ and the structural parameters: *R*(Å) = *R*_0_ − (*dR*/*dP*)^−1^·Δ*ν*. The comparison of the obtained theoretical and known experimental data confirms the efficiency of this technique.

For lattice and intramolecular vibrations active in the Raman spectra, it was obtained that the higher the pressure and the lower the structural parameters, the higher the ν_0_ for each type of vibrations. For the intramolecular vibrations of the ν_4_ type (deformations in the ${\mathrm{CO}}_{3}^{2-}\;\mathrm{plane}$), *v*_1_ (symmetric stretching in the plane), *v*_3_ (asymmetric stretching in the plane), the Gruneisen mode parameter is usually 0.3–0.4 in calcites, dolomites and 0.1–0.2 in aragonites. The second derivative *d*^2^*ν*/*dP*^2^, characterizing the nonlinearity degree of the *ν*(*P*) dependence, is greater for the *v*_3_ mode in carbonates with a heavy cation.

For the low-wavenumber lattice vibrations active in the infrared spectra, it is not possible to describe the dependences of wavenumbers on pressure and structural parameters in linear or quadratic forms. In carbonates with the dolomite and otavite structure, the *dν*/*dP* derivatives are negative, the *da*/*dP* ones are positive; the wavenumbers decrease with the increasing pressure and increase with the decreasing *a* lattice constants. For the *v*_2_ mode (out-of-plane deformation displacements of ${\mathrm{CO}}_{3}^{2-}$ groups), as the pressure increases, a shift of the wavenumbers towards smaller values or nonlinear displacements towards larger ones is observed. The Gruneisen parameter is the size of −0.05 order, and the *da*/*dP* derivative is positive.

The *v*_2_ negative frequency shift is associated with the layered structure of carbonates and strong compressibility anisotropy, when the linear modulus along the *c* axis is 3–4 times less than in the perpendicular direction. For vibrations active in the infrared spectra, when the symmetry allows for cation atom displacements along the *c* axis, with carbon and oxygen atoms in the phase opposite to them, and for vibrations active in the Raman spectra, when of cation atoms displacements are forbidden, anions move towards each other, the *R*_M-O_ distances decrease, and those of *R*_C-O_ increase. With the increasing distances, the overlap population increases and the C-O connection increases. As the *R*_M-O_ distances decrease, metal atom charges decrease, while those of oxygen increase so that their electrostatic interaction energy increases. Thus, in calcite, dolomite and aragonite structures, the *v*_2_ negative shift under pressure is explained by the strengthening of metal-oxygen bonds, and it reduces the deformation force constant outside the plane, as well as by an increase in the cation-oxygen attraction.

The analytical dependences of the vibrational mode wavenumbers on pressure or cell parameters, as obtained in the present study, can be applied in the design of pressure sensors and sensors for the determination of solid solution structure on the base of IRS and RS spectra of carbonate crystals.

## Figures and Tables

**Table 1:** Calculated lattice constants *a* (*a*, *b* for aragonites), *c* and *R*_M-O_ distances between metal atom M and oxygen O (M1-O, M2-O for double carbonates M1M2 (CO_3_)_2_), *R*_C-O_ of carbon C and oxygen O.

Carbonate	*a*(*b*), Å	*c*, Å	*R*_M-O_, Å	*R*_C-O_, Å
MgCO_3_	4.662	15.189	2.123	1.286
ZnCO_3_	4.709	15.130	2.134	1.297
CdCO_3_	4.982	16.616	2.331	1.287
CdMg(CO_3_)_2_	4.814	15.863	2.345, 2.106	1.286
CaMg(CO_3_)_2_	4.838	16.256	2.409, 2.105	1.286
CaZn(CO_3_)_2_	4.856	16.296	2.393, 2.136	1.287
SrCO_3_	5.147, 8.442	6.195	2.684	1.288
PbCO_3_	5.245, 8.572	6.373	2.745	1.290
BaCO_3_	5.367, 8.933	6.685	2.857	1.291

**Table 2:** Equilibrium volume of unit cell per one formula unit *V*_0_/*Z*, volume compression modulus *B*_0_ and its pressure derivative *B*_1_, linear compression moduli along the *a* (*b* for the aragonite structure), *c*-*B*_a_ (*B_b_*), *B_c_* crystallographic axes and the metal-oxygen *B*_M-O_ interatomic distances (M1-O, M2-O for double carbonates M1M2(CO_3_)_2_), and *B*_C-O_ for carbon-oxygen ones.

Carbonate	*V*_0_/*Z*, Å^3^	*B*_0_, GPa	*B*_1_	*B_a_* (*B_b_*), GPa	*B_c_*, GPa	*B_M_*_1*-O*_ (*B_M_*_2-O_), GPa	*B_C-O_*, GPa
MgCO_3_	47.64	107.11	4.28	539	238	326	1749
ZnCO_3_	48.41	123.0	4.91	692	248	384	1768
CdCO_3_	59.53	97.84	3.52	894	159	319	1508
CdMg(CO_3_)_2_	53.06	100.38	4.86	601	189	340 (286)	1589
CaMg(CO_3_)_2_	54.92	88.82	4.33	522	169	269 (276)	1508
CaZn(CO_3_)_2_	55.47	93.39	4.89	587	189	287 (325)	1577
SrCO_3_	67.29	63.27	3.45	397 (328)	125	268	1422
PbCO_3_	71.63	56.21	4.01	403 (496)	100	394	1675
BaCO_3_	80.13	54.39	2.74	477 (434)	88	208	1740

**Table 3:** *E*_0_ zero vibrations energy per formula unit and its *P* pressure derivatives.

Carbonate	*E*_0_, kJ/mol	*dE*_0_*/dP*, kJ/(mol·GPa)	*d*^2^*E*_0_/*dP*^2^, kJ/(mol·GPa^2^)
MgCO_3_	51.201	0.359	−0.006
ZnCO_3_	49.356	0.255	−0.002
CdCO_3_	46.209	0.243	−0.003
CaMg(CO_3_)_2_	49.109	0.308	−0.004
CdMg(CO_3_)_2_	48.759	0.305	−0.005
CaZn(CO_3_)_2_	47.613	0.314	−0.007
SrCO_3_	46.346	0.240	0.017
BaCO_3_	45.211	0.322	−0.003
PbCO_3_	42.774	0.386	−0.008

**Table 4:** Wavenumbers *ν*_0_ (cm^−^^1^) of the intramolecular modes active in the infrared (IRS) and Raman (RS) spectra of the carbonates with the calcite structure, their first *dν*/*dP* (cm^−1^/GPa) and second *d*^2^*ν*/*dP*^2^ (cm^−1^/GPa^2^)) *P* pressure derivatives. The second pressure derivative is given with respect to the *d*^2^*P*/*dν*^2^ (GPa^2^/cm^−1^) wavenumber and the wavenumber derivative—with respect to the *dv*/*da* (cm^−1^/Å) lattice constant.

Mode	Lattice Modes		Internal Modes		
Symmetry	*E_g_*	*E_g_*	*E_g_*(*ν*_4_)	*A_1g_*(*ν*_1_)	*E_g_*(*ν*_3_)
MgCO_3_, RS					
*ν*_0_ *dν/dP* *d*^2^*ν*/*dP*^2^ *d*^2^*P*/*dν*^2^ × 10^−3^ *dv*/*da*	208	323	737	1099	1444
	4.214	5.541	2.90	4.378	5.567
	−0.069	−0.078	−0.072	−0.143	−0.11
	1.47	0.606	6.34	4.42	1.06
	−416	−600	−261	−360	−530
ZnCO_3_, RS								
*ν*_0_ *dν/dP* *d*^2^*ν*/*dP*^2^ *d*^2^*P*/*dν*^2^ × 10^−3^ *dv*/*da*	208	306	723	1105	1457
	3.153	4.611	1.203	2.704	5.426
	0.006	−0.018	0.042	0.001	−0.114
	−0.448	−3.706	−15.73	0.977	12.7
	−470	−653	−231	−398	−649
CdCO_3_, RS								
*ν*_0_ *dν/dP* *d*^2^*ν*/*dP*^2^ *d*^2^*P*/*dν*^2^ × 10^−3^ *dv*/*da*	156	259	713	1093	1395
	2.648	4.945	1.938	3.759	6.925
	0.017	−0.047	−0.005	−0.004	−0.149
	−7.308	5.111	10.77	1.892	9.039
	−452	−736	−306	−603	−918
Symmetry	*E_u_* (T)	*E_u_* (T)	*A*_2*u*_ (T)	*E_u_* (*ν*_4_)	*E_u_(ν*_3_)
MgCO_3_, IRS					
*ν*_0_ *dν/dP* *d*^2^*ν*/*dP*^2^ *d*^2^*P*/*dν*^2^ × 10^−3^ *dv*/*da*	301	344	351	746	1424
	6.628	9.235	9.738	3.128	5.126
	−0.104	−0.144	−0.116	−0.049	−0.087
	0.556	0.282	0.171	2.64	1.023
	−659	−919	−1005	−311	−503
ZnCO_3_, IRS								
*ν*_0_ *dν/dP* *d*^2^*ν*/*dP*^2^ *d*^2^*P*/*dν*^2^ × 10^−3^ *dv*/*da*	212	287	348	735	1447
	1.352	5.761	4.36	1.725	4.95
	0.036	−0.04	0.078	0.04	−0.09
	−6.443	−0.262	−0.475	−2.666	−1.215
	−245	−794	−740	−305	−610
CdCO_3_, IRS					
*ν*_0_ *dν*/*dP* *d*^2^*ν*/d*P*^2^ *d*^2^*P*/*dν*^2^ × 10^−3^ *dv*/*da*	158	267	303	722	1388
	1.616	6.274	6.339	2.295	5.326
	0.035	−0.095	−0.075	0.003	−0.041
	−4.869	0.590	0.422	−0.048	0.339
	−310	−887	−925	−375	−807

**Table 5:** Wavenumbers *ν*_0_ (cm^−^^1^) of intramolecular modes active in the infrared (IRS) and Raman (RS) spectra of carbonates with a dolomite structure, their first *dν/dP* (cm^−1^/GPa) and second *d*^2^*ν*/*dP*^2^ (cm^−1^/GPa^2^) derivatives, with respect to the *P* pressure; the second pressure derivative, with respect to the wavenumber *d*^2^*P*/*dν*^2^ (GPa^2^/cm^−1^), and the wavenumber derivative, with respect to the *dv*/*da* (cm^−1^/Å) lattice constant.

Mode	Lattice Modes				Internal Modes		
Symmetry	*E_g_*	*A_g_*	*E_g_*	*A_g_*	*E_g_*(*ν*_4_)	*A_g_*(*ν*_1_)	*E_g_*(*ν*_3_)
CaMg(CO_3_)_2_, RS							
*ν*_0_ *dν*/*dP* *d*^2^ν/*dP*^2^ *d*^2^*P*/d*ν*^2^ ×10^−3^ *dv*/*da*	175	235	296	336	723	1097	1437
	2.719	1.402	5.397	6.789	2.73	4.596	6.01
	−0.02	0.032	−0.041	−0.09	−0.05	−0.099	−0.109
	−1.114	−7.874	−0.30	0.366	3.473	1.607	0.740
	−279	−171	−552	−670	−260	−260	−573
CdMg(CO_3_)_2_, RS							
*ν*_0_ *dν*/*dP* *d*^2^ν/*dP*^2^ *γ* *d*^2^*P*/d*ν*^2^ × 10^−3^ *dv*/*da*	183	258	286	365	725	1098	1419
	3.516	2.431	5.562	6.094	2.258	3.793	5.437
	−0.071	−0.011	−0.08	−0.04	−0.042	−0.045	−0.07
	1.93	0.94	1.95	1.68	0.31	0.35	0.38
	2.43	0.91	−0.014	0.20	6.685	1.202	0.577
	−383	−293	−629	−726	−249	−437	−622
CaZn(CO_3_)_2_, RS							
*ν*_0_ *dν*/*dP* *d*^2^ν/*dP*^2^ *d*^2^*P*/d*ν*^2^ × 10^−3^ *dv*/*da*	165	233	283	350	719	1095	1416
	3.429	1.796	5.566	7.743	2.622	3.992	5.209
	−0.088	−0.041	−0.072	−0.204	−0.069	−0.059	−0.007
	4.661	0.016	0.602	0.925	8.81	1.5	0.043
	−320	−173	−592	−717	−243	−417	−618
Symmetry	*E_u_*	*A_u_*	*E_u_*	*A_u_*	*E_u_*(*ν*_4_)	*A_u_*(*ν*_1_)	*E_u_*(*ν*_3_)
CaMg(CO_3_)_2_, IRS							
*ν*_0_ *dν*/*dP* *d*^2^ν/*dP*^2^*γ* *d*^2^*P*/d*ν*^2^ × 10^−3^ *dv*/*da*	258	304	337	352	727	1098	1416
	5.26	7.298	9.316	6.647	2.972	4.538	5.6109
	−0.078	−0.168	−0.162	0.022	−0.041	−0.076	−0.112
	0.733	0.702	0.288	−0.069	2.003	1.143	0.966
	−513	−673	−893	−727	−292	−437	−528
CdMg(CO_3_)_2_, IRS							
*ν*_0_ *dν*/*dP* *d*^2^ν/*dP*^2^ *d*^2^*P*/d*ν*^2^ × 10^−3^ *dv*/*da*	236	285	338	354	733	1098	1407
	2.315	2.876	8.981	8.119	2.627	3.964	5.061
	−0.017	−0.064	−0.181	−0.091	−0.022	−0.06	−0.046
	−9.999	4.62	0.378	0.232	2.316	1.401	0.457
	−274	−308	−977	−938	−309	−446	−593
CaZn(CO_3_)_2_, IRS							
*ν*_0_ *dν*/*dP* *d*^2^ν/*dP*^2^ *d*^2^*P*/d*ν*^2^ × 10^−3^ *dv*/*da*	189	242	290	310	724	1094	1400.9
	4.978	3.453	8.139	9.431	2.948	4.089	4.67
	−0.118	−0.084	−0.18	−0.171	−0.066	−0.064	0.013
	1.895	4.171	0.622	0.344	5.179	1.543	−0.128
	−475	−327	−790	−954	−285	−424	−573

**Table 6:** Wavenumbers *ν*_0_ (cm^−1^) of the *v*_4_, *v*_2_, *v*_1_ and *v*_3_ intramolecular modes of carbonates with the aragonite structure; their first *dν*/*dP* (cm^−1^/GPa) and second *d*^2^*ν*/*dP*^2^ (cm^−1^/GPa^2^) *P* pressure derivatives; the second pressure derivative *d*^2^*P*/*dν*^2^ (GPa^2^/cm^−1^), with respect to the wavenumber, and the wavenumber derivative *dv*/*da* (cm^−1^/Å), with respect to the lattice constant.

Mode	*ν*_4_			*ν*_1_	*ν*_3_		
Symmetry	*A_g_*	*B*_1*g*_	*B*_3*g*_	*A_g_*	*A_g_*	*B*_3*g*_	*B*_2*g*_
SrCO_3_, RS							
*ν*_0_ *dν*/*dP* *d*^2^*ν*/*dP*^2^ *d*^2^*P*/d*ν*^2^ × 10^−3^ *dv*/*da*	704	704	708	1080	1453	1453	1567
	4.693	1.546	1.844	3.632	3.649	4.123	4.479
	−0.447	0.012	−0.006	−0.103	−0.008	0.027	0.11
	-	0.968	4.234	2.909	−0.959	−0.236	−0.886
	-	−124	−139	−234	−276	−328	−391
BaCO_3_, RS							
*ν*_0_ *dν*/*dP* *d*^2^*ν*/*dP*^2^ *d*^2^*P*/d*ν*^2^ × 10^−3^ *dv*/*da*	694	696	700	1066	1426	1430	1528
	2.264	1.92	2.275	3.672	4.742	4.739	6.239
	0.039	0.031	0.04	−0.043	−0.349	−0.067	−0.24
	−3.024	−4.593	−3.192	0.768	8.081	0.822	2.967
	−217	−183	−218	−300	−244	−382	−429
PbCO_3_, RS							
*ν*_0_ *dν*/*dP* *d*^2^*ν*/*dP*^2^ *d*^2^*P*/d*ν*^2^ × 10^−3^ *dv*/*da*	680	678	688	1068	1384	1394	1487
	1.006	1.067	2.025	3.261	5.308	2.405	8.416
	0.012	−0.054	−0.067	−0.1	−0.248	0.198	−0.37
	−2.255	112	016	5.402	4.669	−0.698	1.654
	−80.5	−55.9	−122	−200	−288	−266	−467
Symmetry	*B*_2*u*_	*B*_3*u*_	*B*_1*u*_	*B*_1*u*_	*B*_2*u*_	*B*_1*u*_	*B*_3*u*_
SrCO_3_, IRS							
*ν*_0_ *dν*/*dP* *d*^2^*ν*/*dP*^2^ *d*^2^*P*/d*ν*^2^ × 10^−3^ *dv*/*da*	701	708	718	1079	1441	1448	1457
	1.754	2.176	2.418	3.063	4.427	4.162	4.306
	0.00	0.029	0.01	−0.017	0.075	0.067	0.063
	3.915	−1.672	0.340	0.094	−0.584	−0.889	−0.717
	−134	−180	−91	−229	−372	−349	−358
BaCO_3_, IRS							
*ν*_0_ *dν*/*dP* *d*^2^*ν*/*dP*^2^ *d*^2^*P*/d*ν*^2^ × 10^−3^ *dv*/*da*	695	697	707	1066	1426	1420	1431
	2.298	2.471	2.791	3.647	5.441	5.789	5.92
	0.04	0.031	0.037	−0.049	−0.077	−0.33	−0.233
	3.1	−2.038	−1.549	0.912	0.613	4.294	2.27
	−221	−231	−262	−295	−439	−345	−404
PbCO_3_, IRS							
*ν*_0_ *dν*/*dP* *d*^2^*ν*/*dP*^2^ *d*^2^*P*/d*ν*^2^ × 10^−3^ *dv*/*da*	684	682	702	1066	1402	1390	1395
	1.826	1.104	2.196	3.033	2.733	6.143	7.152
	−0.063	0.01	0.013	−0.105	0.208	−0.237	−0.294
	21.0	−0.641	−0.804	7.777	−0.604	2.375	1.999
	−109	−87.0	−170	−180	−295	−355	−405

## Data Availability

Not applicable.
